# Injurious Memories from the COVID-19 Frontline: The Impact of Episodic Memories of Self- and Other-Potentially Morally Injurious Events on Romanian Nurses’ Burnout, Turnover Intentions and Basic Need Satisfaction

**DOI:** 10.3390/ijerph19159604

**Published:** 2022-08-04

**Authors:** Mihaela Alexandra Gherman, Laura Arhiri, Andrei Corneliu Holman, Camelia Soponaru

**Affiliations:** Faculty of Psychology and Education Sciences, Alexandru Ioan Cuza University, Str. Toma Cozma 3, 700554 Iasi, Romania

**Keywords:** potentially morally injurious event (PMIE), turnover intentions, COVID-19 pandemic, basic psychological need satisfaction, nurses, burnout, episodic memories, self-determination theory, memories-as-independent-representations, autobiographical knowledge

## Abstract

Nurses have been frequently exposed to Potentially Morally Injurious Events (PMIEs) during the COVID-19 pandemic. Due to resource scarcity, they both perpetrated (self-PMIEs) and passively witnessed (other-PMIEs) moral transgressions toward the patients, severely violating their moral values. Our study investigated the impact of self- and other-PMIEs on work outcomes by exploring nurses’ episodic memories of these events and the basic psychological need thwarting associated with them. Using a quasi-experimental design, on a convenience sample of 463 Romanian nurses, we found that PMIEs memories were uniquely associated with burnout and turnover intentions, after controlling for socio-demographic characteristics, general basic psychological need satisfaction at work and other phenomenological characteristics. Both self- and other-PMIEs memories were need thwarting, with autonomy and competence mediating their differential impact on burnout, and with relatedness—on turnover intentions. Our findings emphasize the need for organizational moral repair practices, which should include enhancing nurses’ feelings of autonomy, relatedness and competence. Psychological counseling and psychotherapy should be provided to nurses to prevent their episodic memories of PMIEs to be (fully) integrated in autobiographical knowledge, because this integration could have severe consequences on their psycho-social function and occupational health, as well as on the organizational climate in healthcare institutions.

## 1. Introduction

In October 2021, during the fourth wave of the COVID-19 pandemic, Romania hit record spikes in infections and death tolls, with the second highest per capita COVID-19 death rate in the world [1]. Severely understaffed, underfinanced and with a poorly developed infrastructure as compared to the other European Union (EU) states [2], the Romanian healthcare system reached maximum capacity for accommodating both incoming cases and the bodies of the deceased [3]. Healthcare workers from all medical specialties were faced with dramatic pressures to ensure proper patient care, with officials asking the EU for help with medical supplies and the relocation of surgeries, through the EU Civil Protection Mechanism [4]. This type of crisis was shown to be a fertile ground for potentially morally injurious events (PMIEs) [5,6,7,8,9].

Nurses’ exposure to PMIEs has soared during the COVID-19 pandemic in Romania and all around the world, due to the ethical challenges posed by resource scarcity [5,6,7,8,9]. PMIEs are most often not acknowledged immediately after being experienced, but rather upon recall, which means that their impact on psycho-social functioning is dependent upon a person’s memory of the event [8,10]. Although the effects of nurses’ memories of PMIEs during the COVID-19 pandemic on some well-being indicators have been explored before [10], we aim to widen the scope of this incipient line of research and further investigate their impact on nurses’ lives and careers. Drawing from Self-Determination Theory (SDT), recent research on moral memories [11] and moral injury [5], our study aims to investigate how memories of work-related PMIEs influence nurses’ burnout and turnover intentions after at least six months from exposure. We distinguish between self- and other-PMIEs, in line with recent recommendations [5,12,13], and explore the phenomenological characteristics of the resulting memories. Using a quasi-experimental design, we also investigate the differential impact of nurses’ exposure to the two types of PMIEs on the two outcomes, to test whether the basic psychological need-thwarting experienced during recall can have a unique impact on nurses’ current burnout and turnover intentions.

### 1.1. Episodic Memories of PMIEs

Initially studied in war veterans, PMIEs are events which entail “perpetrating, failing to prevent, bearing witness to, or learning about acts that transgress deeply held moral beliefs and expectations” [14] (p. 697). While moral injury is always caused by exposure to PMIEs, not all PMIE experiences lead (only) to moral injury, which is why research needs to better differentiate between the two [12,13].

Another conceptual differentiation insufficiently investigated distinguishes between perpetration- and betrayal-based PMIEs (i.e., self- and other-PMIEs) [12,13]. Self-PMIEs are moral violations perpetrated by the person under perceived environmental constraint, whereas other-PMIEs are moral violations perpetrated by others and passively witnessed by the person. These constructs are best viewed as correlated, but distinctive, because of their different effects on the emotional health of healthcare providers [5,15]. Only exposure to self-PMIEs predicted levels of anxiety, PTSD and burnout above the clinical cutoffs, while also being significantly associated with disengagement [5]. However, both types of PMIE exposure were associated with scores surpassing the clinical cutoff for depression, worse quality of life and burnout in samples including nurses [5,15].

An important characteristic of PMIE exposure is its acknowledgement after the occurrence of the event itself [8]. This would imply that the effects of exposure to PMIEs on psycho-social functioning and well-being are dependent upon people’s episodic memories of the events. The prevailing theories on the role of autobiographical episodic memories support a top-down approach, which posits that the conceptual self regulates the long-term memory encoding of episodic memories (i.e., the memories-as-proxy hypothesis) [16,17]. In other words, we recall episodic memories insofar as they support our established views and opinions about ourselves (i.e., autobiographical knowledge) [18,19]. In this perspective, episodic memories serve to contextualize certain aspects of the self in vivid, sensory detail [20]. Thus, they cannot orient or regulate behavior independently, but rather guided by the conceptual self [18]. In this view, episodic memories of PMIEs would not be retained in autobiographical knowledge, since they conflict with nurses’ conceptual selves, which are morally good [21,22]. However, self-PMIEs were associated with higher levels of re-experiencing, guilt and self-blame compared to life-threating traumatic events leading to PTSD, suggesting that episodic memories of PMIEs are retained [23]. Moreover, although they are perceived as abnormal and dramatic as related to the self and the self-schemas, they threaten personal integrity and may cause the loss of individual meaning-making elements [24]. This would suggest that their re-experiencing may not be regulated by the existing conceptual self for consolidating existing autobiographical knowledge [18,19].

A novel line of research on episodic memories, the memories-as-independent-representations hypothesis [25] can help us shed light on this phenomenon. Supporting a bottom-up approach, episodic memories were shown to sometimes influence our behaviors, psycho-social functioning and well-being independently of our self-concepts [25,26,27]. This is true especially for memories of traumatic events, when it is particularly difficult to reconcile existing identities with the new information about the self, extracted from the episodic memories [28]. For instance, the new information in a memory of a self-PMIE would portray the nurse as immoral in her profession, capable of harming patients or their families. This information about them as *persons* would dramatically conflict with their *moral self-concepts* [21], which could lead to a difficult integration of the episodic memory in autobiographical knowledge, at times lasting for more than two years [18,29]. However, these episodic memories can still guide actions and behaviors, even before being fully integrated [25].

Episodic memories which are not (yet) integrated in autobiographical knowledge may be associated with goal structures [20] through macroscopic goals (i.e., human innate psychological needs to which we are naturally oriented) [25]. The role of episodic memories would then be to encode events relevant to one’s goal system but diverging from autobiographical knowledge and the conceptual self. They would impact subjective experiences and behavior through their goal-affective components, such as the level of basic psychological need satisfaction [25,26,27,30,31,32,33,34,35]. Autonomy, competence and relatedness are three psychological needs which have been consistently reported cross-culturally to support well-being, growth and health at all ages [36,37].

Basic psychological needs can range from being satisfied to being thwarted (i.e., frustrated) in a certain context, such as the workplace [36], or during a specific event and its memory [25,26,27,30,31,32,33,34,35]. When autonomy is satisfied, people feel volitional and authentic in their actions, opposed to when autonomy is thwarted, when actions are performed under external and unwelcomed pressure, and feel disconnected from the person’s own will and goals. When competence is satisfied, people feel effective and efficient, whereas when competence is thwarted, they feel inadequate and may perceive themselves as failures. Satisfied relatedness translates into feelings of mutual connectedness and caring, while relatedness-thwarting may be experienced as feeling neglected, disregarded and disrespected. When satisfied, all three lead to well-being, both together and separately; when thwarted, they lead to illbeing [36,37].

Although we may not formulate specific goals to satisfy or avoid thwarting these needs, most of our goals have these superordinate objectives, irrespective of the content of the conceptual self [25]. Need-satisfying/thwarting episodic memories are closely linked to our goal systems, to inform us quickly about what situations should be avoided or approached based on our previous experiences [34,38,39]. There is a strong body of research showing that need-satisfaction/thwarting is a key goal-affective component of episodic memories, able to better predict outcomes than most other phenomenological memory characteristics (e.g., valence, intensity, vividness, voluntary and involuntary recall, visual detail, sense of reliving, importance and centrality to the self) [25,26,27,30,31,32,33,34,35].

Past studies have investigated the effects of exposure to self- and other-PMIEs on health and well-being outcomes after a significant amount of time had elapsed since the incident, in healthcare workers and in war veterans e.g., [5,15]. Thus, the effects they found could be attributed to *memories* of PMIEs rather than *exposure* to them. However, the instruments they employed (i.e., scales measuring whether exposure had occurred) and the differences in the amount of time having passed from exposure to data collection prevent us from drawing precise conclusions concerning the role of their episodic memories in the respective outcomes [5,10,12,13], since episodic memories from the same time period have to be recalled in detail during data collection to explore this topic [11,25,26,27,30,31,32,33,34,35].

The results of a previous study we conducted supported a unique association between Romanian nurses’ episodic memories of PMIEs and burnout, work motivation, work satisfaction, moral learning and adaptive performance, mediated by autonomy-thwarting [10]. Thus, memories of PMIEs were more autonomy-thwarting than memories of severe moral transgressions (SMTs), leading to increased burnout and decreased work motivation, work satisfaction, moral learning and adaptive performance. Nevertheless, the study did not control for nurses’ general need-satisfaction at work, which could have biased those findings, in line with previous research [25,27], which also indicates that the predictive power of need-thwarting should be greater than other phenomenological memory characteristics for work-related well-being outcomes.

Our previous study compared memories of PMIEs to memories of SMTs, which functioned as a control group, because the moral transgressions were similar in severity, but different in the degree to which personal or professional moral values were violated through (in)action. However, it did not distinguish between self- and other-PMIEs, nor did it explore the role of competence and relatedness-thwarting. Although moral stressors were associated with feelings of decreased personal and professional competence [40,41], self-PMIEs are instances of immoral action, whereas other-PMIEs, of immoral inaction. According to the omission bias, immoral inactions are judged as less harmful and associated with less blame compared to immoral actions [42]. Since nurses define their competence according to their code of ethics, with moral values central to their professional identity [21], their need for competence could be more thwarted when they perpetrate the perceived immoral act than when they witness it without interfering.

In contrast, nurses’ need for relatedness could be more thwarted in memories of other-PMIEs, because they depict instances of organizational betrayal, perpetrated by their superiors [5,12,13]. Following the same rationale, their autonomy could be more thwarted in memories of other-PMIEs, which are accompanied by disrespect towards nurses’ autonomy and their exclusion from medical decision-making processes by their peers [43,44,45]. Although self-PMIEs are also characterized by feelings of diminished autonomy [40,41], they are also associated with more guilt than other-PMIEs [12], which could indicate a stronger sense of personal responsibility and, consequently, less autonomy-thwarting.

### 1.2. Burnout and Turnover Intentions in Nurses during the COVID-19 Pandemic

Burnout is a syndrome generated by chronic exposure to work-related stress, manifesting through exhaustion, cynicism, negative work attitudes and decreased professional efficacy [46]. Nurses’ burnout has increased during the COVID-19 pandemic, although results about its association with moral injury are mixed [47,48]. One study indicated that exposure to PMIEs was associated with burnout in nurses, although the notions of “moral distress”, “moral injury” and “PMIEs” were used interchangeably [49], against recommendations for more conceptual clarity in the field [7]. We previously found that nurses’ memories of PMIEs could uniquely contribute to burnout through autonomy-thwarting, but did not control for other relevant predictors, such as age, sex, education, work experience, weekly hours of work and marital status [50,51].

Considered the best predictor of turnover behavior [52], turnover intentions represent a conscious and deliberate willfulness to quit one’s job, being “the last in a sequence of withdrawal cognitions, a set to which thinking of quitting and intent to search for alternative employment also belong” [53] (p. 262). The COVID-19 pandemic has increased nurses’ turnover intentions [54], which were already high compared to other professions, even before the pandemic [55]. This increase was attributed to the diversifying of their predictors, among other factors, which called for more in-depth investigation in this area [54,56,57]. In military personnel and teachers during the COVID-19 pandemic, exposure to PMIEs increased turnover intentions as well [13,57,58]. Episodic memories were shown to predict turnover intentions in members of educational organizations through their need-satisfying/thwarting components [25]. Relevant socio-demographic predictors include age, sex, education, work experience, weekly hours of work and marital status [59,60,61,62].

### 1.3. Present Study

To sum up, our hypotheses are the following:

**H1.**  *Basic psychological need-thwarting in episodic memories of PMIEs is uniquely associated with burnout and turnover intentions, independent of general need-satisfaction at work and socio-demographic characteristic (i.e., age, sex, education, work experience, weekly hours of work and marital status).*

**H2.**  *Basic psychological need-thwarting in episodic memories of PMIEs is associated more strongly with burnout and turnover intentions than other phenomenological characteristics of the memories (i.e., emotional valence and intensity, voluntary and involuntary recall, vividness, reliving, visual detail, importance and centrality to the self).*

**H3.**  *The need for competence is more thwarted in memories of self-PMIEs compared to other-PMIEs, whereas the needs for autonomy and relatedness are more thwarted in memories of other-PMIEs than of self-PMIEs.*

**H4.**  *Memories of self-PMIEs are associated with higher burnout and turnover intentions compared to memories of other-PMIEs.*

**H5.**  *The differences in strength of association between memories of self- and other-PMIEs and, respectively, burnout and turnover intentions are mediated by basic psychological need-thwarting.*

## 2. Materials and Methods

### 2.1. Participants

A convenience sample of 463 Romanian nurses working in hospitals selected through snowballing techniques participated in our study, conducted from April to May 2022. The data was collected after a fourth wave of the COVID-19 pandemic, which had a catastrophic impact on Romanian healthcare institutions, with infection and mortality reaching unprecedented peaks of more than 500 daily deaths and approximately 20,000 daily new cases, in a country with 19 million inhabitants [63]. Considering the unpreparedness of the medical system to manage this crisis and based on results from prior waves of the pandemic in this context [64], we expected that nurses in most health specialties could have suffered from exposure to PMIEs and we strived to include in our sample a diverse range of specialty to increase representativeness.

Initially, we contacted 650 nurses via e-mail and/or phone and invited them to participate in this research. Their contact data had been obtained in previous research conducted by the authors, when participants consented to being contacted again for research purposes. We also asked the nurses to forward the invitation to other peers who met the inclusion criteria we had set for this study: having worked as a nurse in a hospital during the COVID-19 pandemic for more than six months. Out of the 650 nurses contacted, 492 expressed their interest in participating. Another 82 nurses contacted us via e-mail/telephone after being invited by their peers. Upon randomization in the two experimental conditions, we sent links for Google Forms to 574 participants, with 287 participants in each condition. Of the participants, 37 withdrew from the study, 27 from the self-PMIE and 10 from the other-PMIE condition. Furthermore, 11 participants were eliminated because they failed the attention check (4 from the self-PMIE condition and 7 from the other-PMIE condition). Another 33 participants from the self-PMIE condition and 26 from the other-PMIE condition were eliminated because they did not recall PMIEs, according to their answers on the Moral Injury Events Scale. Hence, our final sample comprised 463 nurses, with 240 in the other-PMIE condition (51.8%) and 223 in the self-PMIE condition (48.2%), from varied specialties (Table 1).

Our research followed the ethical guidelines from the Declaration of Helsinki and received approval from the ethics committee of our faculty. All participants were over 18 and agreed to the informed consent, which instructed them about their voluntary involvement and data confidentiality issues. Because of the sensitive nature of the data collected (episodes of PMIEs at their workplace), we assured participants that their anonymity would be kept and none of their data would be made public or shared with anyone other than the two main investigators (i.e., the first two authors). We adopted this policy due to our participants raising issues that they may face drastic consequences if their identities were discernable. The data collected was securely stored by the two first authors for statistical analysis. As a reward for their participation, five cash prizes of RON 100 were offered through a draw.

### 2.2. Procedure and Instruments

The study was self-paced. Upon consulting and agreeing with the informed consent, participants filled in socio-demographic information concerning age, sex, education, weekly hours of work and current specialty. Participants filled in the scales measuring burnout, turnover intentions and general psychological need-satisfaction at work, with previous research recommending their administration before memory recall, so that answers were not biased by the mood changes caused by memories [31].

*Burnout* was assessed with the Maslach Burnout Inventory, validated on the Romanian population [65]. A final score (α = 0.925) was computed by summing up participants’ scores on the three psychological parameters assessed: *emotional exhaustion* (EE; nine items; α = 0.956), *depersonalization* (DP; five items; α = 0.931) and *personal accomplishment* (PA; eight items; α = 0.945). Responses were provided on a seven-point Likert-type scale, from 0—“Never” to 6—“Everyday”.

*Nurses’ turnover intentions* were measured with the three-item scale from the Michigan Organizational Assessment Questionnaire [66] (e.g., “I often think about leaving the organization”). Responses were provided on a seven-point Likert-type scale, from 1—“Strongly disagree” to 7—“Strongly agree”. Alpha was 0.899.

*Work need-satisfaction* was assessed with the eighteen-item Work-Related Basic Need Satisfaction scale [67], which measured the degree to which the three basic psychological needs of *autonomy* (e.g., “The tasks I have to do at work are in line with what I really want to do.”), *competence* (e.g., “I really master my tasks at my job.”) and *relatedness* (e.g., “At work, I feel part of a group.”) were experienced as satisfied at work in general. Responses were provided on a seven-point Likert-type scale. The instrument measures need-satisfaction at the level of the self-concept and should be differentiated from the evaluation of need-satisfaction in a specific work episode, as detailed for the work-related memory assessment presented below. Indices averaging the items for each need and, respectively, for all three needs, were computed, a common procedure in Self-Determination Theory (SDT) research [36]. Cronbach’s alpha coefficients for the indices were as follows: for autonomy, 0.943; for competence, 0.961, and, for relatedness, 0.951. For the entire scale, Cronbach’s alpha was 0.865.

Participants were then asked to recall episodic memories of self- and other-PMIEs, according to the experimental condition to which they had been randomly assigned, after being presented with definitions and examples of the two constructs, as explained in Appendix A. Then, we administered the nine-item Moral Injury Events Scale (MIES) modified to assess PMIEs among healthcare workers during the COVID-19 pandemic [68] (e.g., “I acted in a way that violated my own moral code or values in this instance.”). The scale was tested and used on Romanian healthcare workers [6]. Answers ranged from 1—“Strongly Agree” to 6—“Strongly Disagree”. To assess whether memories were perceived as PMIEs, we dichotomized the total scores, with responses of “Moderately Agree” to “Strongly Agree” on any of the nine items coded as exposure to a PMIE [68], excluding participants not recalling PMIEs.

All participants were asked to provide their moral judgement on the events recalled (“How morally wrong was your behavior in this instance?”), from 1—“Slightly Morally Wrong” to 7—“Very Morally Wrong” [11]. To verify our experimental manipulation, we asked all participants the extent to which they perceived themselves as witnesses and, respectively, as perpetrators during the events on scales ranging from 1—“Strongly Disagree” to 7—“Strongly Agree”.

Next, we asked participants to rate the level of need-satisfaction they experienced during their work memories, because past studies showed a large correlation between participants’ and independent judges’ ratings [32] (Study 1). Each need was evaluated with two items on a seven-point Likert scale, ranging from −3 (Strongly disagree) to +3 (Strongly agree), with 0—do not agree nor disagree or not applicable: autonomy (e.g., “I felt free to do things and to think how I wanted”), competence (e.g., “I felt that my reaction was adequate”) and relatedness (e.g., “I felt connected to one or more people”) [25,27,32]. Then, individual scores for each basic need were computed and averaged, with higher scores reflecting higher need-satisfaction. Scores above zero represent a need satisfying memory, while scores less than or equal to of zero represent a need-thwarting memory. The items and the procedure were extensively used in past research e.g., [25,26,27,30,31,32,33,34,35], which usually combined the assessment of the three psychological needs into a second-order factor, in line with SDT e.g., [69,70,71,72]. For the purposes of our study, we assessed need-satisfaction both separately and for the three needs combined. Hence, we conducted a confirmatory factor analysis for the six items, which showed a good fit for a three-factor model, χ^2^(6) = 17.9, *p* = 0.006, χ^2^/df = 2.98, CFI = 0.995, TLI = 0.987, RMSEA = 0.066, 90% CI [0.03; 0.10], SRMR = 0.014, with standardized estimates of factor loadings ranging from 0.89 to 0.91. These findings show that the items properly discriminate between the three needs, while also forming a construct. Therefore, we averaged the scores for the three needs in a need satisfaction index, with a Cronbach’s alpha of 0.921. The reliability for the three dimensions was also assessed: Cronbach’s alpha for Autonomy = 0.88; Cronbach’s alpha for Competence = 0.897; Cronbach’s alpha for Relatedness = 0.892.

We then asked participants two questions about their emotional experiences, targeting the valence and intensity of their emotions: “as you remember the event now, how positive or negative are your emotions, from 1 = very negative to 7 = very positive?”; “as you remember the event now, how intense are your emotions, from 1 = not at all intense to 7 = very intense?” [11]. To reflect negative emotional experiences, scores were reversed. Next, to assess the frequency of voluntary and involuntary retrieval [11,73,74,75]: “Since it happened, how often have you willfully thought about the event in your mind or talked about it?” (1 = *Never*, 7 = *Very often*); “Since it happened, has the memory of the event suddenly popped up in your thoughts by itself—that is, without your having attempted to remember it?” (1 = *Never*, 7 = *Very often*).

We also investigated other phenomenological characteristics of the memories recalled, with items adapted from the Memory Characteristics Questionnaire (MCQ) [76] and the Autobiographical Memory Questionnaire (AMQ) [77], following [78]. Thus, we assessed, with one item each, the vividness (“Overall, how vivid is your memory of the event?” 1 = *Very vague* to 7 = *Very vivid*), visual detail (“To what extent does this remembered event involve visual detail?” 1 = *No details* to 7 = *Many details*) and sense of reliving (“While remembering the event, do you feel as though you are mentally reliving it?” 1 = *Not at all* to 7 = *Completely*), along with the personal importance (“How important is the event to you personally? (It involves an important episode in your life” 1 = *Not at all important* to 7 = *Very important*) and centrality of the events (“Is the event in your memory a central part of your life story?” 1 = *Not at all central* to 7 = *Very central*).

We used the attention check applied by [11]: “Do you feel that you paid attention, avoided distractions and took the survey seriously?” Participants were told that their responses would not impact their prize draw or their opportunity to take part in future research. Answers ranged from: 1—“No, I was distracted”; 2—“No, I had trouble paying attention”; 3—“No, I did not take this study seriously”; 4—“No, something else affected my participation negatively”; 5—“Yes”. We eliminated from our data analysis participants who answered 1, 2, 3 or 4.

## 3. Results

Data analyses were carried out in jamovi 2. To check our experimental manipulation, we ran three Independent Sample *t*-Tests to assess differences between the two experimental groups according to the perceived moral severity of the recalled PMIE, the perceived role of witness and, respectively, perpetrator played by the participant. Results showed no differences according to moral severity, as well as significant differences in terms of the two roles, supporting the validity of the experimental manipulation (Table A1).

To verify participant randomization in the two experimental groups, we assessed with Independent Sample *t*-Tests and Chi-Square Tests of Association differences according to participants’ age, sex, education, work experience, weekly hours of work, marital status and general need-satisfaction at work. We found no significant differences, except for marital status, for which there were more single participants in the self-PMIE group and more married participants in the other-PMIE group (see Table 1 and Table A1). Consequently, this variable was treated as an independent variable.

### 3.1. Participants’ Characteristics and Differences between Them According to Outcome Variables

Socio-demographic differences in outcomes of interest were assessed with Welch’s Independent Sample *t*-Tests and One-Way ANOVAs, due to violations of the assumption of equal variances and unequal sample sizes (Table A2). For this purpose, we stratified “age” and “work experience”.

Participants with less work experience (i.e., less than or equal to 10 years) experienced significantly less *general basic psychological need-satisfaction at work* than participants with more work experience (i.e., between 11 and 36 years). Similarly, younger participants (i.e., 21–30 years old) experienced less general basic psychological need-satisfaction at work as compared to participants aged from 31–40 years old (*t*(216) = −7.2, *p* < 0.001) and as compared to participants aged from 41–57 years old (*t*(255) = −7.37, *p* < 0.001). Participants who worked 36 h a week experienced more general basic psychological need-satisfaction than both participants who worked 48 h a week (*t*(329) = 6.26, *p* < 0.001) and participants who worked 60 h a week (*t*(124.8) = 5.88, *p* < 0.001) (Table A2).

Participants who were single experienced significantly more *burnout* as compared to their married counterparts, similar to participants with less work experience, who reported more burnout than participants with more work experience. This trend was mirrored by the effects of participants’ age, with those aged from 21 to 30 reporting more burnout than those aged from 31 to 40 (*t*(269) = 9.26, *p* < 0.001) and more than those aged from 41 to 57 (*t*(240) = 5.62, *p* < 0.001). Participants who worked 36 h a week reported less burnout than participants who worked 48 h a week (*t*(248) = −8.33, *p* < 0.001) and less than participants who worked 60 h a week (*t*(140) = −7.01, *p* < 0.001). We also looked at differences according to the three dimensions of burnout (EE, DP, PA) and found the same pattern of results for EE, but no significant effect of marital status for PA and DP (Table A2).

*Turnover intentions* were stronger for single participants as compared to married participants, and weaker for participants who worked 36 h a week as compared to participants who worked 48 h a week (*t*(255) = −2.68, *p* = 0.021) and as compared to those who worked 48 h a week (*t*(166) = −2.955, *p* = 0.010) (Table A2).

Finally, to explore differences in outcomes of interest according to participants’ specialty, we ran a series of One-Way ANOVAs, after removing the one participant we had from the Dentistry specialization, to have sufficient observations for every category of the grouping variable. Since the assumptions about homogeneity of variances and normality of the distributions were met, we used Fisher’s coefficients with Tukey post-hoc tests. We only found significant differences in burnout between different specialties (*F*(14, 447) = 1.82, *p* = 0.033), with no significant differences in post-hoc tests. Given the unequal group sizes and their high number in relation to the total sample size, this result should be interpreted with caution.

### 3.2. Correlational Analyses

Pearson’s correlations were computed to assess the associations between the outcome variables and the characteristics of the memories (Table A3). Significant positive associations were found between participants’ turnover intentions and frequency of voluntary and involuntary retrieval, emotional intensity and valence, vividness, visual detail, sense of reliving, importance and centrality to the self of memories of PMIEs, and, respectively, both burnout and turnover intentions. In contrast, the higher the turnover intentions and the higher the burnout, the lower the general basic psychological need-satisfaction at work, and the lower the basic psychological need-satisfaction during the recalled PMIE, a trend registered for autonomy, competence and relatedness. As expected, significant correlations were found between the majority of the phenomenological characteristics of the PMIE memories, as well as between them and basic psychological need-satisfaction during the recalled PMIE (Table A3). Participants’ sex was significantly correlated with relatedness-thwarting (*r =* 0.104, *p* = 0.026) only, with men’s memories of PMIEs being associated with more relatedness-thwarting than females.

### 3.3. The Influence of Basic Psychological Need-Satisfaction in Memories of PMIEs on Nurses’ Burnout and Turnover Intentions

To test H1 and H2, we conducted a series of hierarchical regressions (Table 2). Correlational analyses showed that age and work experience were highly associated (*r* = 0.81, *p* < 0.001); to avoid collinearity violations, we only kept “age” in the analyses, the demographic with the highest correlations with burnout (*r* = −0.22, *p* < 0.001) and turnover intentions (*r* = −0.094, *p* = 0.043) [25].

Results have confirmed the unique contribution of basic psychological need-thwarting during the recalled PMIE to explaining additional variance for both burnout and turnover intentions, when controlling for socio-demographic characteristics and, respectively, for other phenomenological characteristics of the memories. Prior to adding basic psychological need-thwarting during the recalled PMIE to the model, weekly hours at work, age, marital status and general need-satisfaction at work were significantly related to burnout, of which only weekly hours and general need-satisfaction at work remained significant in Step 2. Turnover intentions were significantly predicted by marital status and general need satisfaction at work, of which only the latter remained significant upon adding basic psychological need-thwarting during the recalled PMIE to the model.

For phenomenological characteristics, burnout was significantly predicted by involuntary retrieval, emotional intensity and importance to the self before adding basic psychological need-thwarting during the recalled PMIE to the model, when only emotional intensity remained a significant predictor. Turnover intentions were significantly predicted by involuntary retrieval and emotional intensity before adding basic psychological need-thwarting during the recalled PMIE to the model, when both relationships were rendered insignificant.

### 3.4. Differences in Phenomenological Characteristics of Memories and Psycho-Social Work Outcomes According to the Type of PMIE Recalled (Self- vs. Other-PMIE)

To test H3 and H4, and to explore other differences in phenomenological characteristics according to the type of PMIE recalled (self- vs. other-PMIE), we conducted a series of Independent Sample *t*-Tests (See Table 3). Autonomy and competence were more thwarted in memories of self-PMIEs compared to memories of other-PMIEs, whereas relatedness is more thwarted in memories of other-PMIEs rather than self-PMIEs. However, the means of the three basic psychological needs were below zero for both memories of self- and other-PMIEs, indicating that they are need-thwarting memories [25,27,32]. Memories of self-PMIEs were associated with higher burnout than memories of other-PMIEs, with significant differences for all its three dimensions: EE, DP and PA.

Memories of self-PMIEs were experienced in more visual detail and as more vivid, emotionally negative and intense, important and central to the self, being retrieved more often involuntarily and voluntarily and associated with a greater sense of reliving as compared to memories of other-PMIEs. Additionally, participants rated both types of memories as rather important and central to their selves (means between 4.77 and 5.24 out of a possible 7—indicating moderately to considerably important).

### 3.5. The Mediating Role of Basic Need-Satisfaction in the Relationship between Type of PMIE and Work Outcomes

To test H4, we conducted two mediation analyses in jamovi, module jAMM, with the type of memory (self- vs. other-PMIE) as the independent variable, the three basic psychological needs as mediators, and burnout and turnover intentions as dependent variables (Table 4). Results showed that basic need-satisfaction fully mediated the association between the type of memory and turnover intentions, with significant indirect effects for autonomy and relatedness. Thus, autonomy and competence were more thwarted, and relatedness was more satisfied in memories of self-PMIEs than in memories of other-PMIEs. The more thwarted the needs of autonomy and relatedness, the higher the turnover intentions. This suggests that the main differences between the two types of memories regarding nurses’ intentions might be explained by the differences in the thwarting of autonomy and relatedness.

For burnout, we found that its relationship with the type of memory (self- vs. other-PMIE) was partially mediated by relatedness and competence. Thus, autonomy and competence were more thwarted, and relatedness was more satisfied in memories of self-PMIEs than in memories of other-PMIEs. The more thwarted the needs of competence and relatedness, the higher the burnout. This indicates that the main differences between the effects of the two types of memories on burnout reside in the extent to which the needs for relatedness and competence were thwarted (Table 4). Supplementary mediation analyses indicated that competence and relatedness partially mediated the relationship between the type of memory and EE, and that the relationships between the type of memory, DP and PA were partially mediated by relatedness (Table A4). This would suggest that the differences in personal accomplishment, depersonalization and emotional exhaustion between memories of self- and other-PMIEs were associated with differences in relatedness and in competence-thwarting, with the latter being true only for emotional exhaustion.

## 4. Discussion

This study aimed to investigate the effects of nurses’ episodic memories of PMIEs during the COVID-19 pandemic on their burnout and turnover intentions, according to basic psychological need-thwarting, phenomenological characteristics and socio-demographic characteristics. Using a quasi-experimental design, we differentiated between memories of self- and other-PMIEs in our analyses, while also clearly delineating between exposure to PMIEs and moral injury, in line with past recommendations [5,12,13]. Overall, our results suggest that nurses’ exposure to both self- and other-PMIEs may still have a detrimental impact on their occupational health after at least six months, mainly according to the degree of basic psychological need-thwarting associated with their episodic memories of the events.

Autobiographical episodic memories are the building blocks for our identity [16,17,18,19,20]. Though not all episodic memories are retained in autobiographical knowledge [18], we remember those that best illustrate who we are (i.e., our conceptual selves). When episodic memories severely contradict our autobiographical knowledge and our identities, we tend to forget or distort them to better fit our narratives about ourselves and our lives [18]. However, not all conflicting episodic memories are forgotten or distorted by the conceptual self [25]; some, especially the more traumatic ones, are retained [29], either to be eventually integrated into autobiographical knowledge [79,80], or because they are intimately related with our goal systems [16,28]. Thus, they have the capacity of directing our behavior [81,82] and affecting our psycho-social health and functioning [25,26,27,30,32,33,34,35]. Our findings suggest that nurses’ memories of self- and other-PMIEs may be closely linked to the three macroscopic, innate goals of human beings (i.e., autonomy, competence, relatedness) [36] and, thus, be retained in long-term memory, potentially for subsequent integration, since they were rated as moderately to considerably important and central to the self [25]. At any rate, they were uniquely associated with nurses’ burnout and turnover intentions, indicating that they may function as behavior guides and affect nurses’ occupational health.

If the memories of PMIEs having occurred during the COVID-19 pandemic are to be integrated in nurses’ autobiographical knowledge by nurses, this would dramatically change their work identities [25,27], with negative consequences on their psychological health and on the healthcare system. Previous studies found that a high rate of nurses and other healthcare providers have been exposed to PMIEs during this pandemic e.g., [5,6,7,8,9,47,48,49,50,54,56,57,62,64,68]. Moral values and work ethics are central to the identity of nurses, guiding them in their professional activity and providing meaning to their work [21]. Should these identities be modified by their episodic memories of PMIEs, they would see themselves as capable of perpetrating or idly witnessing immoral acts in relation to patients [16,18]. Given that self-concept regulates behavior, they could repeat these actions in the future in more or less similar circumstances [11], which would have deleterious consequences on patientcare. Alienation from fellow providers and their occupation may also ensue, especially for other-PMIEs, which severely thwarted their need for relatedness, affecting the organizational climate [83,84]. Finally, given the incoherence between their current self-representations and beliefs and their memories of PMIEs, the integration of the latter could lead to greater posttraumatic symptoms [85].

Our findings support an ongoing attempt at integrating memories of PMIEs into autobiographical knowledge, with participants reporting moderate to considerable voluntary and involuntary retrieval of memories of PMIEs, reliving, vividness, visual detail, importance and centrality to the self [25]. These phenomenological characteristics were significantly more heightened for memories of self-PMIEs than for memories of other-PMIEs, differences not studied so far, but which could be attributed to the omission bias [42]. This is in line with theoretical perspectives arguing that frequent reliving and voluntary and involuntary retrieval could constitute attempts at integrating episodic memories into autobiographical knowledge, especially for traumatic memories, such as PMIEs [79,80]. The implication of these findings is that healthcare organizations should take action as soon as possible and provide nurses with psychological counseling and psychotherapy aimed at recontextualizing their memories of these events before they are fully integrated into autobiographical knowledge [12].

We also found that episodic memories of PMIEs are need-thwarting, with memories of self-PMIEs being associated with significantly less autonomy and competence as compared to other-PMIEs, and with the latter being associated with more relatedness-thwarting than the former. Although we expected that autonomy would be more thwarted in memories of other-PMIEs, this was not confirmed by our results. A possible explanation would be that the environmental/external constraints perceived by nurses when perpetrating self-PMIEs were stronger than we had anticipated. This could also be the result of their attempting to reconciliate the information about the self from the memories of self-PMIEs with their morally good identities [21,25]. Intentionality is an important predictor of retaining episodic memories of moral transgressions, perpetrated by the self and by others, with more unintentional transgressions being judged less severely and, thus, more easily forgotten [86]. In conclusion, since self-PMIEs are associated with more severe clinical manifestations and with more guilt [5,12], nurses may try to reconstruct their memories to better fit their conceptual selves by magnifying the autonomy-thwarting they felt during the events.

Regardless of the mechanisms at play in these instances, the need-thwarting in both types of memories could significantly impact their work identities as well. As such, they could perceive themselves as less competent and autonomous subsequent to self-PMIEs, which could redefine their professional roles as nurses, along with other parameters of psychological health and well-being [25,27]. Our findings indicate that at least burnout and turnover intentions are affected by this. Moreover, the relatedness-thwarting associated with memories of other-PMIEs may undermine their trust in the organization and in their superiors, which could lead to either helplessness or even disobedience when it comes to following physicians’ recommendations [87]. Feeling disconnected from one’s peers would also affect patient care by impairing the necessarily good communication between the members of the medical team in charge of patient care [7,36].

Currently, there are few studies documenting the differential effects of exposure to self- and other-PMIEs [5,12,13]. Distinguishing between self- and other-PMIEs is necessary to document their distinct pathological outcomes, better understanding reported symptoms, choosing clinical courses of treatment and estimating responses to treatment [12]. Our study showed that higher burnout and turnover intentions associated with memories of self-PMIEs compared to memories of other-PMIEs are in line with past research on burnout [5]. The same differences were found for EE, DP and AP. Moreover, our results indicate that the differential impact of self- and other-PMIE memories on the two work outcomes are mediated by basic psychological need-thwarting, a novel contribution to the literature, to the best of our knowledge.

Specifically, memories of self-PMIEs were associated with higher burnout due to more competence-thwarting, whereas memories of other-PMIEs were associated with higher burnout due to relatedness-thwarting, a pattern of differences we found only with EE. This is in line with previous research showing that EE in teachers was higher when they felt less competent or insufficiently connected with their students [88]. It also supports past research showing how central moral values are for nurses’ professional identities, suggesting that feeling incompetent at their jobs can bring about feelings of strain and the depletion of emotional resources [21,89]. Moreover, it supports the importance of an inclusive climate in healthcare organizations, suggesting that feeling disconnected from one’s peers may have similar effects as feeling incompetent at one’s job. Interestingly, only relatedness-thwarting mediated the relationship between the type of memory and PA. This would imply that, upon witnessing other-PMIEs, the thwarted connectedness led to feelings of loss of professional efficacy, which would mean that nurses no longer expected to be able to perform their jobs according to their moral standards in a work environment allowing for such severe moral violations.

This interpretation is also corroborated by the fact that lower relatedness in memories of other-PMIEs was associated with higher turnover intentions, emphasizing that nurses might consider leaving a workplace or a profession where they cannot connect to their peers on moral grounds. Our participants were more likely to consider leaving their job due to autonomy-thwarting in the case of self-PMIEs. This is in line with previous research, which showed that perceived autonomy support at work decreases burnout, compassion fatigue and turnover intentions while also increasing work satisfaction, organizational identification, innovative behavior, work engagement and job performance [90,91,92,93,94,95].

Our findings also contribute to the growing body of knowledge supporting the hypothesis that episodic memories may remain accessible and functional even when/if they are not integrated into autobiographical knowledge (i.e., the memories-as-independent-representations perspective) [25]. According to this perspective, and corroborated by our results, episodic memories may fulfill the function of encoding events relevant to one’s goal system but conflict with data previously deposited into the autobiographical knowledge and the conceptual self [25]. Hence, memories of PMIEs may act as independent representations and guide actions and outcomes regardless of the conceptual self, which could be very dangerous for nurses as well as for other professional categories affected in this manner by the COVID-19 pandemic.

Concerning socio-demographic characteristics, our findings suggest that younger and more inexperienced nurses are more likely to be dissatisfied with their work in terms of their basic psychological need-satisfaction, as well as nurses who worked for more hours a week. This confirms previous findings showing that nurses’ basic need-satisfaction at work does not vary with level of education, marital status or sex, but that it does decrease with experience [96,97]. We also found that participants who were single, less experienced, younger or spending more time at work experienced more burnout. Previous research on the effects of age on nurses’ burnout has rendered mixed results, with findings suggesting that younger age significantly predicted higher emotional exhaustion, depersonalization, but not personal accomplishment [51]. However, we found younger age to predict all three dimensions of burnout. A recent review of predictors of nurses’ turnover intentions showed that the COVID-19 pandemic may have increased nurses’ turnover intentions. The intentions were dependent upon nurses’ age and work experience, with younger and less experienced nurses being more likely to consider leaving the profession [62]. Another predictor was the time spent caring for patients, with more time being associated with an increased turnover intention. Marital status and sex have traditionally predicted both turnover intentions and burnout [59,60,61]. Our findings, however, suggest that only being single and weekly hours at work significantly impact nurses’ turnover intentions in our sample, in the sense that nurses who spend less time at work are less likely to consider leaving their jobs.

Our research is not without limitations. First, our design was quasi-experimental because we did not have a control group with which to compare our findings. Future research should verify our results by including a control group of nurses exposed to traumatic events without moral implications during the COVID-19 pandemic or nurses diagnosed with PTSD. Second, our research was cross-sectional, which means that we cannot draw any definitive conclusions regarding causality. Future studies should verify our findings longitudinally. Our sample of participants was not representative for the population from which it was drawn, although we strived to include nurses from several specialties. Finally, future research should also investigate the content of the memories of self- and other-PMIEs thematically. Although we had hoped that our participants would allow us to use their data this way, this was not the case, given the sensitive nature of the information. Hopefully, researchers in other geo-cultural contexts may accomplish this in future studies.

Our results indicate that unique events of exposure to PMIEs can have long-term consequences on nurses’ psycho-social functioning and well-being. Future research should contribute to the development of more effective training programs to adequately prepare nurses to deal with ethically challenging events. Early intervention programs for nurses showing mental health symptoms as a consequence of PMIEs should also be devised. Healthcare institutions should then provide them with access to the necessary psychotherapies to address these issues, according to the type of PMIE to which they have been exposed. For self-PMIEs, research has shown the efficiency of strategies to ease guilt and shame, such as contextualization, nonjudgmental acceptance of emotions, cultivating openness to receiving and providing forgiveness (to others and to self) and conciliatory actions [98,99]. For other-PMIEs, treatments focused on moral and/or spiritual repair could prove efficient [100,101,102]. However, since the consequences of exposure to PMIEs are not reduced to intrapsychic conflicts, psychotherapy may not be enough for recovery. Healthcare organizations would have to make an affirmative community effort to comprehend and reintegrate workers exposed to PMIEs during the pandemic, and to accept shared responsibility for the occurrence of these traumatic events [12].

## 5. Conclusions

Our study investigates the effects of nurses’ exposure to self- and other-PMIEs during the COVID-19 pandemic on burnout and turnover intentions after at least six months post-exposure by exploring their episodic memories of these events. To the best of our knowledge, this is the first study which addresses the impact of recalling PMIEs on work outcomes in nurses, despite the fact that PMIEs are usually acknowledged post-exposure. We also operate clear distinctions between exposure to PMIEs and moral injury and, respectively, between self- and other-PMIEs. Our results suggest that episodic memories of PMIEs have a unique association with burnout and turnover intentions through basic psychological need-thwarting, when controlling for socio-demographic variables, general basic psychological need-satisfaction at work and other phenomenological characteristics of the memories. While memories of self-PMIEs are overall more impairing for nurses’ psycho-social functioning, the high relatedness-thwarting associated with memories of other-PMIEs is notable, indicating that different strategies for moral repair should be employed organizationally to address this issue. Finally, our results suggest that nurses’ episodic memories of PMIEs could be undergoing the process of integration into autobiographical knowledge, which would dramatically affect nurses’ work identities, behavior and psychological health, as well as the healthcare landscape in general. Consequently, there should be an urgent interest to recontextualize nurses’ memories of PMIEs through counseling and psychotherapy.

## Figures and Tables

**Table 1:** Participants’ characteristics.

	Experimental Condition				Total (*N = 463*)	
	Other-PMIE (*N = 240*)		Self-PMIE (*N = 223*)			
**Specialty**						
Dentistry	0	0%	1	0.45%	1	0.22%
Emergency	16	6.67%	24	10.76%	40	8.64%
Gastroenterology	10	4.17%	3	1.35%	13	2.81%
Hematology	6	2.5%	2	0.9%	8	1.73%
Intensive Care Units	8	3.33%	23	10.31%	31	6.7%
Infectious Diseases	15	6.25%	15	6.73%	30	6.48%
Internal Medicine	16	6.67%	13	5.83%	29	6.26%
Internal Medicine Chronic	15	6.25%	8	3.59%	23	4.97%
Neurology	23	9.58%	17	7.62%	40	8.64%
Obstetrics Gynecology	10	4.17%	8	3.59%	18	3.89%
Oncology	35	14.58%	24	10.76%	59	12.74%
Palliation	30	12.5%	29	13%	59	12.74%
Pneumology	13	5.42%	12	5.38%	25	5.4%
Psychiatry	18	7.5%	15	6.73%	33	7.13%
Radiology	0	0%	6	2.69%	6	1.3%
Surgery	25	10.42%	23	10.31%	48	10.37%
**Age**						
*M* ± *SD*	38.8 ± 7.92		37.7 ± 9.11		38.2 ± 8.52	
Range	23–54		21–57		21–57	
**Sex**						
Female	211	87.92%	188	84.30%	399	86.18%
Male	29	12.08%	35	15.70%	64	13.82%
**Education**						
Post-Secondary studies	216	90%	206	92.38%	422	91.14%
Bachelor’s degree	10	4.17%	13	5.83%	23	4.97%
Master’s degree	14	5.83%	4	1.79%	18	3.89%
**Marital status**						
Single	99	41.25%	124	55.61%	223	48.16%
Married	141	58.75%	99	44.39%	240	51.84%
**Weekly hours of work**						
*M* ± *SD*	45.8 ± 8.52		47 ± 7.05		46.4 ± 7.86	
Range	36–60		36–60		36–60	
**Work experience (years)**						
*M* ± *SD*	13.1 ± 7.72		11.6 ± 8.63		12.4 ± 8.20	
Range	1–34		0.5–36		0.5–36	

**Table 2:** Hierarchical multiple regressions of work outcomes on need-satisfaction in work-related PMIEs memories.

	Burnout				Turnover Intentions			
	Step 1		Step 2		Step 1		Step 2	
	Β ^a^		Β ^a^		Β ^a^		Β ^a^	
				**H1**				
Weekly hours	0.26	***	0.19	***	0.07		−0.02	
Age	−0.09	*	−0.05		−0.01		0	
Marital status	−0.1	*	−0.02		−0.27	***	−0.17	***
WBPNS	−0.28	***	−0.15	***	−0.18	***	−0.02	
MBPNS			−0.42	***			−0.53	***
F	35.9	***	55.3	***	16.6	***	47	***
df	4, 458		5, 457		4, 458		5, 457	
R2	0.239		0.377		0.126		0.339	
Adj R2	0.232		0.37		0.119		0.332	
R2 change	0.138	***			0.213	***		
				**H2**				
Voluntary retrieval	0.03		0.05		0.06		0.07	
Involuntary retrieval	0.14	*	0.04		0.15	*	0.04	
Vividness	0		0		0		0	
Visual detail	0.02		0		0.09		0.07	
Reliving	0		0		0.01		0.03	
Importance to the self	0.13	*	0.08		0.05		0	
Centrality to the self	0.05		0.05		0		0	
Emotional intensity	0.22	***	0.14	**	0.13	*	0.04	
MBPNS			−0.47	***			−0.5	***
F	10.9	***	27.9	***	8.66	***	26.83	***
df	8, 454		9, 453		8, 454		9, 453	
R2	0.161		0.357		0.132		0.348	
Adj R2	0.147		0.344		0.117		0.335	
R2 change	0.195	***			0.215	***		

Note: ^a^ Standardized coefficients. WBPNS = General level of basic psychological need-satisfaction at work; MBPNS = Basic psychological need-satisfaction during the recalled PMIE. *** *p* < 0.001, ** *p* < 0.01, * *p* < 0.05.

**Table 3:** Differences between memories of self-PMIEs and memories of other-PMIEs regarding their basic psychological need-thwarting, burnout, turnover intentions and other phenomenological characteristics.

	Other-PMIE (*N* = 240)		Self-PMIE (*N* = 223)		Independent Sample *t*-Tests				
	*M*	*SD*	*M*	*SD*	*t*		*df*	Effect Size ^b^	
Autonomy	−0.35	1.05	−1.29	1.03	9.71	***	461	0.9	
Competence	−0.39	1.13	−1.31	1.24	8.34	***	461	0.8	
Relatedness	−1.1	1.21	−0.46	0.93	−6.46 ^a^	***	446	−0.6	
Emotional valence	4.55	1.27	5.06	1.47	−3.97 ^a^	***	440	−0.4	
Emotional intensity	5.38	1.21	5.73	1.14	−3.21	**	461	−0.3	
Voluntary retrieval	5.28	1.11	5.63	1.19	−3.33 ^a^	***	452	−0.3	
Involuntary retrieval	4.54	1.25	4.97	1.51	−3.34 ^a^	***	432	−0.3	
Vividness	4.95	1.16	5.32	1.4	−3.03 ^a^	**	432	−0.3	
Visual detail	4.94	1.12	5.31	1.28	−3.32 ^a^	***	442	−0.3	
Reliving	4.85	1.12	5.2	1.36	−2.99 ^a^	**	431	−0.3	
Importance to self	4.77	1.21	5.24	1.37	−3.9 ^a^	***	444	−0.4	
Centrality to self	4.81	1.22	5.2	1.34	−3.31	**	461	−0.3	
Burnout	53.7	12.54	75.2	11.92	−18.89	***	461	−1.76	
Emotional exhaustion	21.6	8.19	30.9	7.64	−12.59	***	461	−1.17	
Depersonalization	11.7	4.87	17.5	4.98	−12.56	***	461	−1.17	
Personal accomplishment	20.4	6.93	26.9	6.93	−10.03	***	461	−0.93	
Turnover intentions	13.3	2.95	14.2	3.02	−3.5	***	461	−0.33	

Note: ^a^ Levene’s test is significant (*p* < 0.05), suggesting a violation of the assumption of equal variances. Hence, Welch’s *t*-tests are reported. ^b^ Cohen’s *d* coefficient for effect size. *** *p* < 0.001, ** *p* < 0.01.

**Table 4:** Basic psychological needs as mediators of the relationships between type of memory (self vs. other), turnover intentions and burnout.

	Effects of Type of Memory ^a^			Unique Effect of Mediators			Indirect Effect				
								BP 95% CI			
	b	β		b	β		b	LL	UL	β	
	**Turnover intentions**										
Autonomy ^b^	−0.94	−0.41	***	−1.09	−0.43	***	1.02	0.56	1.56	0.18	***
Competence ^b^	−0.92	−0.36	***	0.12	0.05		−0.11	−0.49	0.3	−0.02	
Relatedness ^b^	0.65	0.29	***	−0.68	−0.27	***	−0.44	−0.76	−0.18	−0.08	**
Direct effect	0.5	0.09									
Total effect	0.97	0.16	***								
	**Burnout**										
Autonomy ^b^	−0.94	−0.41	***	0.81	0.06		−0.76	−2.78	1.04	−0.02	
Competence ^b^	−0.92	−0.36	***	−2.17	−0.17	**	2	0.57	3.47	0.06	**
Relatedness ^b^	0.65	0.29	***	−5.41	−0.38	***	−3.49	−4.93	−2.24	−0.11	***
Direct effect	23.76	0.75	***								
Total effect	21.51	0.66	***								

Note: ^a^ Type of memory: Contrast = 1-0, 1 = self-PMIE. ^b^ Mediators. *** *p* < 0.001, ** *p* < 0.01.

## Data Availability

The data presented in this study are partially available upon request from the corresponding author. The data are not publicly available due to ethical constraints created by the sensitive topic investigated, which precludes us from sharing the content of the memories of moral transgressions recalled by participants, as well as information based on which participants could be identified.

## Appendix A

Experimental procedure.

We presented all participants with definitions and examples for self- and other-PMIEs. The examples reflected severe moral transgressions, operationalized as the magnitude of the harmful effects on patients [103]. The moral violations were devised according to Brüggemann et al. [104]. The definition of self-PMIEs provided to the participants was: “events or action during which you felt as both a moral victim and a moral transgressor, when you did something that you felt was morally wrong not because you wanted to, but because you felt as if you did not have a choice” [9].

The definition of others-PMIEs provided to the participants was: “events or action during which you felt as both a moral victim and a moral transgressor, when you witnessed something that you felt was morally wrong and failed to act or speak out not because you wanted to, but because you felt as if you did not have a choice” [9].

The example for self-PMIEs was: “Laura is a nurse at a hospital in Romania. During the 4th wave of the COVID-19 pandemic, the beds in the ICU were all occupied and she had to care for several patients with COVID-19 in the ER. Four patients were rapidly deteriorating, all of them desperately needing access to ventilators. None were available, and Laura had to decide to start manual ventilation on one of them. The physicians on call were not answering, and patients’ oxygen saturations were dropping quickly. In the spur of the moment, she started the procedure on the youngest patient, a 12-year-old child. Until other nurses could join her, the oldest patient of the four died. Laura felt incredibly guilty for not having saved his life”.

The example for others-PMIEs was: “Laura is a nurse at a hospital in Romania. During the 4th wave of the COVID-19 pandemic, the beds in the ICU were all occupied and she had to care for several patients with COVID-19 in the ER. Four patients were rapidly deteriorating, all of them desperately needing access to ventilators. None were available, and the physician on call had to decide to start manual ventilation on two of them. He told Laura to start the procedure on the youngest patient, a 12-year-old child, while he proceeded to do the same on a 26-year-old female. Until other medical staff could join them, the oldest patient of the four died. Laura felt incredibly guilty for not having saved his life, since, in her opinion, his condition was the most critical of the four, but felt that she could not have disobeyed the doctor’s order”.

The examples referenced work-related situations to prepare the participants to recall memories central to their main work activity—caring for patients.

Then, participants in the self-PMIEs condition received the following instruction: “Please describe a personal memory of a specific event related to your work during the COVID-19 pandemic which you consider a self-PMIE, as defined and exemplified above. Select a memory significant to you which is at least six-months-old, and which often comes to your mind. This memory should be of the most morally wrong thing you have done during the pandemic with harmful consequences for a patient, under environmental constraint. Describe, in a general fashion, what happened, where it happened, who you were with (if anyone), and how you and other people reacted. Please remember we are not interested in the identities of anybody involved, so feel free to use phrases such as ‘a colleague’, ‘a boss’, ‘a patient’ and other generic denominators. What is important to us is for you to remember specific details, not for us to know them. Describe your role and what have been the consequences of your reaction or of your actions during this event. Please provide enough details so that we can fully understand what happened, as if you were telling a story to someone. We would also like to assure you that the content of your memories will not be shared with anybody outside of the two first authors and it will not be used in our analyses”. Participants in the others-PMIE condition received the same instruction, with the first two sentences modified as such: “Please describe a personal memory of a specific event related to your work during the COVID-19 pandemic which you consider an other-PMIE, as defined and exemplified above. Select a memory significant to you which is at least six-months-old, and which often comes to your mind. This memory should be of the most morally wrong thing you have witnessed during the pandemic with harmful consequences for a patient, against which you wanted to speak out or take action, but you felt you could not”.

## Appendix B

**Table A1:** Differences between the two experimental groups according to perceived moral severity, role, age, work experience, sex, general need-satisfaction at work, weekly work hours, sex, education, marital status.

	Self-PMIE	Other-PMIE				
	*M* ± *SD*	*M* ± *SD*	*t*	*df*	*p*	Cohen’s *d*
Moral severity	6 ± 0.83	5.94 ± 0.81	−0.82	461	0.411	−0.08
Witness role	2.74 ± 1.47	5.51 ± 1.14	22.55 ^a^	418	<0.001	2.11
Perpetrator role	5.38 ± 1.11	2.72 ± 1.42	−22.49 ^a^	448	<0.001	−2.08
Age	37.69 ± 9.11	38.77 ± 7.9	1.35 ^a^	441	0.177	0.13
Work experience	11.67 ± 8.71	13.13 ± 7.72	1.9 ^a^	444	0.058	0.18
WBPNS	3.58 ± 0.74	3.71 ± 0.75	1.908	461	0.057	0.18
Weekly hours of work	47.03 ± 7.05	45.75 ± 8.52	−1.77 ^a^	455	0.078	−0.16
			χ^2^	*df*	*p*	*N*
Sex (Male/Female)			1.27	1	0.261	463
Education (PS/B/M)			5.57	2	0.062	463
Marital status (Single/Married)			9.54	1	0.002	463

Note: ^a^ Levene’s test was significant (*p* < 0.05), suggesting a violation of the assumption of equal variances. Therefore, Welch’s *t*-test was reported. PS = Post-Secondary Studies; B = Bachelor’s Degree; M = Master’s Degree; WBPNS = General level of basic psychological need-satisfaction at work.

**Table A2:** Socio-demographic differences according to perceived general need-satisfaction at work, emotional exhaustion, depersonalization, personal accomplishment, burnout and turnover intentions.

Characteristics	*N*	*M* ± *SD*	Welch’s *t*/*F*	Cohen’s *d*/η²	Games-Howell Post-Hoc ^a^
WBPNS					
**Sex**			*t*(98.1) = 0.68, *p* = 0.498	0.08	
Male	64	3.7 ± 0.61			
Female	399	3.64 ± 0.77			
**Marital status**			*t*(460) = −1.13, *p* = 0.257	−0.11	
Single	223	3.61 ± 0.74			
Married	240	3.68 ± 0.75			
**Work experience (years)**			*t*(457) = −4, *p* < 0.001	−0.37	-
0.5–10	231	3.51 ± 0.7			
11–36	232	3.78 ± 0.77			
**Age (years)**			*F*(2,271) = 33.98, *p* < 0.001	0.108	G1 < G2 ***, G1 < G3 ***
21–30 (G1)	100	3.18 ± 0.6			
31–40 (G2)	178	3.74 ± 0.64			
41–57 (G3)	185	3.81 ± 0.81			
**Education**			*F*(2,28.6) = 0.24, *p* = 0.788	0.001	-
PS	422	3.65 ± 0.74			
B	23	3.55 ± 0.93			
M	18	3.71 ± 0.54			
**Weekly hours**			*F*(2,165) = 25.49, *p* = < 0.001	0.114	G1 > G2 ***, G1 > G3 **
36 (G1)	135	4.02 ± 0.73			
48 (G2)	256	3.55 ± 0.63			
60 (G3)	72	3.3 ± 0.88			
**Emotional exhaustion**					
**Sex**			*t*(85.6) = 1.13, *p* = 0.262	0.15	-
Male	64	27.22 ± 8.99			
Female	399	25.85 ± 9.21			
**Marital status**			*t*(461) = 2.89, *p* = 0.004	0.27	-
Single	223	27.3 ± 8.92			
Married	240	24.86 ± 9.3			
**Work experience (years)**			*t*(422) = 4.93, *p* < 0.001	0.46	-
0.5–10	231	28.1 ± 7.45			
11–36	232	23.99 ± 10.25			
**Age (years)**			*F*(2,273) = 13.68, *p* < 0.001	0.038	G1 > G2 ***, G1 > G3 ***
21–30 (G1)	100	29.44 ± 6.86			
31–40 (G2)	178	25.19 ± 7.22			
41–57 (G3)	185	25.01 ± 11.33			
**Education**			*F*(2,28.9) = 0.6, *p* = 0.557	0.002	-
PS	422	26.07 ± 9.3			
B	23	26.78 ± 8.47			
M	18	24.28 ± 7.31			
**Weekly hours**			*F*(2,172) = 27.79, *p* = <0.001	0.11	G1 < G2 ***, G1 < G3 ***
36 (G1)	135	21.37 ± 8.61			
48 (G2)	256	27.64 ± 8.29			
60 (G3)	72	29.1 ± 10.13			
**Depersonalization**					
**Sex**			*t*(94.6) = 1.15, *p* = 0.252	0.14	-
Male	64	15.17 ± 4.85			
Female	399	14.4 ± 5.82			
**Marital status**			*t*(461) = 1.7, *p* = 0.089	0.16	-
Single	223	14.97 ± 5.48			
Married	240	14.07 ± 5.87			
**Work experience (years)**			*t*(448) = 3.77, *p* < 0.001	0.35	-
0.5–10	231	15.49 ± 5.1			
11–36	232	13.52 ± 6.09			
**Age (years)**			*F*(2,273) = 8.54, *p* < 0.001	0.028	G1 > G2 ***, G1 > G3 **
21–30 (G1)	100	16.32 ± 4.71			
31–40 (G2)	178	14.04 ± 5.11			
41–57 (G3)	185	13.96 ± 6.49			
**Education**			*F*(2,28.9) = 2.39, *p* = 0.109	0.007	-
PS	422	14.56 ± 5.75			
B	23	15.22 ± 5.16			
M	18	12.22 ± 4.6			
**Weekly hours**			*F*(2,184) = 11.27, *p* = < 0.001	0.049	G1 < G2 ***, G1 < G3 ***
36 (G1)	135	12.57 ± 5.75			
48 (G2)	256	15.42 ± 5.52			
60 (G3)	72	14.88 ± 5.39			
**Personal accomplishment**					
**Sex**			*t*(86.3) = 0.01, *p* = 0.994	0	-
Male	64	23.53 ± 7.41			
Female	399	23.52 ± 7.69			
**Marital status**			*t*(461) = 1.43, *p* = 0.154	0.13	-
Single	223	24.05 ± 7.36			
Married	240	23.04 ± 7.88			
**Work experience (years)**			*t*(427) = 2.57, *p* = 0.011	0.24	-
0.5–10	231	24.43 ± 6.42			
11–36	232	22.62 ± 8.62			
**Age (years)**			*F*(2,268) = 8.66, *p* < 0.001	0.029	G1 > G2 ***, G1 > G3 **
21–30 (G1)	100	25.98 ± 6.39			
31–40 (G2)	178	22.9 ± 6.4			
41–57 (G3)	185	22.79 ± 9.01			
**Education**			*F*(2,28.5) = 2.3, *p* = 0.119	0.007	-
PS	422	23.4 ± 7.69			
B	23	26.22 ± 6.05			
M	18	22.94 ± 8.04			
**Weekly hours**			*F*(2,174) = 21.3, *p* = < 0.001	0.092	G1 < G2 ***, G1 < G3 ***
36 (G1)	135	20.11 ± 7.55			
48 (G2)	256	24.46 ± 6.94			
60 (G3)	72	26.6 ± 8.04			
**Burnout**					
**Sex**			*t*(90.8) = 1.07, *p* = 0.286	0.14	-
Male	64	65.92 ± 14.64			
Female	399	63.77 ± 16.54			
**Marital status**			*t*(456) = 2.92, *p* = 0.004	0.27	-
Single	223	66.32 ± 14.64			
Married	240	61.97 ± 17.46			
**Work experience (years)**			*t*(359) = 5.37, *p* < 0.001	0.5	-
0.5–10	231	68.02 ± 10.77			
11–36	232	60.13 ± 19.6			
**Age (years)**			*F*(2,298) = 48.46, *p* < 0.001	0.061	G1 > G2 ***, G1 > G3 ***
21–30 (G1)	100	71.74 ± 6.84			
31–40 (G2)	178	62.14 ± 10.4			
41–57 (G3)	185	61.77 ± 22.28			
**Education**			*F*(2,29) = 2.08, *p* = 0.143	0.006	-
PS	422	64.04 ± 16.49			
B	23	68.22 ± 12.14			
M	18	59.44 ± 15.47			
**Weekly hours**			*F*(2,173) = 40.36, *p* = < 0.001	0.16	G1 < G2 ***, G1 < G3 ***
36 (G1)	135	54.05 ± 15.75			
48 (G2)	256	67.52 ± 14.11			
60 (G3)	72	70.57 ± 16.36			
**Turnover intentions**					
**Sex**			*t*(88.7) = 1.83, *p* = 0.071	0.24	-
Male	64	14.34 ± 2.8			
Female	399	13.65 ± 3.04			
**Marital status**			*t*(457) = 6.37, *p* < 0.001	0.59	-
Single	223	14.63 ± 2.64			
Married	240	12.92 ± 3.11			
**Work experience (years)**			*t*(451) = 1.09, *p* = 0.276	0.1	-
0.5–10	231	13.9 ± 2.78			
11–36	232	13.59 ± 3.24			
**Age (years)**			*F*(2, 267) = 2.85, *p* = 0.060	0.012	-
21–30 (G1)	100	13.55 ± 2.74			
31–40 (G2)	178	14.16 ± 2.85			
41–57 (G3)	185	13.45 ± 3.28			
**Education**			*F*(2,28.1) = 0.91, *p* = 0.413	0.004	-
PS	422	13.8 ± 3.03			
B	23	13.04 ± 2.84			
M	18	13.33 ± 3.03			
**Weekly hours**			*F*(2,187) = 5.17, *p* = 0.007	0.023	G1 < G2 *, G1 < G3 **
36 (G1)	135	13.06 ± 3.19			
48 (G2)	256	13.95 ± 2.95			
60 (G3)	72	14.31 ± 2.72			

Note: ^a^ Only significant Post-Hoc Tests are summarily presented. *** *p* < 0.001, ** *p* < 0.01, * *p* < 0.05. PS = Post-Secondary Studies; B = Bachelor’s Degree; M = Master’s Degree; WBPNS = General level of basic psychological need-satisfaction at work.

**Table A3:** Correlations between phenomenological qualities of autobiographical memories, general basic psychological nee- satisfaction at work, burnout and turnover intentions.

		1		2		3		4		5		6		7		8		9		10		11		12		13		14		15	
1	Turnover	-																													
2	Burnout	0.31	***	-																											
3	WBPNS	−0.22	***	−0.4	***	-																									
4	Autonomy	−0.56	***	−0.61	***	0.36	***	-																							
5	Competence	−0.5	***	−0.61	***	0.37	***	0.87	***	-																					
6	Relatedness	−0.45	***	−0.24	***	0.31	***	0.59	***	0.61	***	-																			
7	MBPNS	−0.56	***	−0.55	***	0.39	***	0.92	***	0.94	***	0.82	***	-																	
8	E.valence	0.24	***	0.26	***	−0.22	***	−0.28	***	−0.22	***	−0.17	***	−0.25	***	-															
9	E.intensity	0.28	***	0.3	***	−0.26	***	−0.3	***	−0.25	***	−0.24	***	−0.29	***	0.6	***	-													
10	Voluntary.R	0.28	***	0.26	***	−0.19	***	−0.22	***	−0.23	***	−0.13	**	−0.22	***	0.46	***	0.46	***	-											
11	Involuntary.R	0.31	***	0.31	***	−0.2	***	−0.3	***	−0.29	***	−0.25	***	−0.31	***	0.52	***	0.49	***	0.58	***	-									
12	Vividness	0.24	***	0.24	***	−0.16	***	−0.24	***	−0.2	***	−0.14	**	−0.21	***	0.43	***	0.43	***	0.54	***	0.61	***	-							
13	Visual detail	0.27	***	0.23	***	−0.13	**	−0.23	***	−0.22	***	−0.12	**	−0.21	***	0.4	***	0.4	***	0.54	***	0.49	***	0.5	***	-					
14	Reliving	0.22	***	0.21	***	−0.12	*	−0.2	***	−0.18	***	−0.12	*	−0.19	***	0.44	***	0.38	***	0.5	***	0.48	***	0.48	***	0.47	***	-			
15	Importance	0.23	***	0.27	***	−0.09		−0.23	***	−0.23	***	−0.13	**	−0.22	***	0.4	***	0.34	***	0.47	***	0.4	***	0.47	***	0.47	***	0.56	***	-	
16	Centrality	0.2	***	0.24	***	−0.08		−0.17	***	−0.2	***	−0.13	**	−0.19	***	0.45	***	0.35	***	0.41	***	0.43	***	0.43	***	0.43	***	0.51	***	0.53	***

Note: WBPNS = General level of basic psychological need-satisfaction at work; MBPNS = Basic psychological need-satisfaction during the recalled PMIE; E.valence = emotional valence; E.intensity = emotional intensity; Voluntary.R = voluntary retrieval; Involuntary.R = involuntary retrieval. *** *p* < 0.001, ** *p* < 0.01, * *p* < 0.05.

**Table A4:** Basic psychological needs as mediators in the relationship between type of memory (self vs. other) and emotional exhaustion, depersonalization and personal accomplishment.

	Effects of Type of Memory ^a^			Unique Effect of Mediators			Indirect Effect				
								BP 95% CI			
	b	β		b	β		b	LL	UL	β	
	**Emotional exhaustion**										
**Autonomy ^b^**	−0.94	−0.41	***	1.06	0.13		−1	−2.37	0.22	−0.06	
**Competence ^b^**	−0.92	−0.36	***	−1.34	−0.23	**	1.51	0.5	2.58	0.08	**
**Relatedness ^b^**	0.65	0.29	***	−2.63	−0.32	***	−1.69	−2.58	−0.93	−0.09	***
**Direct effect**	10.48	0.57	***								
**Total effect**	9.29	0.51	***								
	**Depersonalization**										
**Autonomy ^b^**	−0.94	−0.41	***	−0.03	−0.01		0.03	−0.79	0.8	0	
**Competence ^b^**	−0.92	−0.36	***	−0.58	−0.13		0.53	−0.12	1.18	0.05	
**Relatedness ^b^**	0.64	0.29	***	−0.87	−0.18		−0.56	−1.05	−0.12	−0.05	*
**Direct effect**	5.75	0.51	***								
**Total effect**	5.75	0.51	***								
	**Personal accomplishment**										
**Autonomy ^b^**	−0.94	−0.41	***	−0.22	−0.03		0.21	−1	1.35	0.01	
**Competence ^b^**	−0.92	−0.36	***	0.05	0		−0.04	−1.05	0.87	0	
**Relatedness ^b^**	0.64	0.29	***	−1.91	−0.28	***	−1.23	−1.99	−0.59	−0.08	***
**Direct effect**	7.54	0.5	***								
**Total effect**	6.47	0.42	***								

Note: ^a^ Type of PMIE: Contrast = 1-0, 1 = self-PMIE. ^b^ Mediators. *** *p* < 0.001, ** *p* < 0.01, * *p* < 0.05.

## References

[1] Euronews with AP (2021). COVID-19: Romania in Eye of Storm with Record Infections and Deaths. https://www.euronews.com/2021/10/19/romania-in-eye-of-covid-storm-with-death-rate-among-world-s-highest.

[2] Popescu M., Stefan O.M., Stefan M., Valeanu L., Tomescu D. (2022). ICU-Associated Costs during the Fourth Wave of the COVID-19 Pandemic in a Tertiary Hospital in a Low-Vaccinated Eastern European Country. Int. J. Environ. Res. Public Health.

[3] Reuters Romania’s COVID-19 Deaths Hit Record as Intensive Care Beds Run Out. 19 October 2021. https://www.reuters.com/world/europe/romania-reports-record-number-deaths-covid-19-infections-2021-10-19/.

[4] Furlong A. (2021). Romania Suspends Surgeries, Asks EU for Help as It Battles Coronavirus Wave. https://www.politico.eu/article/romania-surgeries-eu-coronavirus-help/.

[5] Dale L.P., Cuffe S.P., Sambuco N., Guastello A.D., Leon K.G., Nunez L.V., Bhullar A., Allen B.R., Mathews C.A. (2021). Morally Distressing Experiences, Moral Injury, and Burnout in Florida Healthcare Providers during the COVID-19 Pandemic. Int. J. Environ. Res. Public Health.

[6] Maftei A., Holman A.C. (2021). The prevalence of exposure to potentially morally injurious events among physicians during the COVID-19 pandemic. Eur. J. Psychotraumatol..

[7] Riedel P.-L., Kreh A., Kulcar V., Lieber A., Juen B. (2022). A Scoping Review of Moral Stressors, Moral Distress and Moral Injury in Healthcare Workers during COVID-19. Int. J. Environ. Res. Public Health.

[8] Rushton C.H., Thomas T.A., Antonsdottir I.M., Nelson K.E., Boyce D., Vioral A., Swavely D., Ley C.D., Hanson G.C. (2021). Moral Injury and Moral Resilience in Health Care Workers during COVID-19 Pandemic. J. Palliat. Med..

[9] Williamson V., Murphy D., Greenberg N. (2020). COVID-19 and experiences of moral injury in front-line key workers. Occup. Med..

[10] Gherman M.A., Arhiri L., Holman A.C., Soponaru C. (2022). The Moral Impact of the COVID-19 Pandemic on Nurses’ Burnout, Work Satisfaction and Adaptive Work Performance: The Role of Autobiographical Memories of Potentially Morally Injurious Events and Basic Psychological Needs. Int. J. Environ. Res. Public Health.

[11] Stanley M.L., Cabeza R., Smallman R., De Brigard F. (2021). Memory and Counterfactual Simulations for Past Wrongdoings Foster Moral Learning and Improvement. Cogn. Sci..

[12] Griffin B.J., Purcell N., Burkman K., Litz B.T., Bryan C.J., Schmitz M., Villerme C., Walsh J., Maguen S. (2019). Moral injury: An Integrative Review. J. Trauma. Stress.

[13] Michaud K., Suurd Ralph C., Connick-Keefer S.J.A. (2021). Operational stressors, psychological distress, and turnover intentions: The impact of potentially morally injurious experiences. Mil. Psychol..

[14] Litz B.T., Stein N., Delaney E., Lebowitz L., Nash W.P., Silva C., Maguen S. (2009). Moral injury and moral repair in war veterans: A preliminary model and intervention strategy. Clin. Psychol. Rev..

[15] Nieuwsma J.A., O’Brien E.C., Xu H., Smigelsky M.A., Meador K.G., VISN 6 MIRECC Workgroup, HERO Research Program (2022). Patterns of Potential Moral Injury in Post-9/11 Combat Veterans and COVID-19 Healthcare Workers. J. Gen. Intern. Med..

[16] Conway M., Pleydell-Pearce C. (2000). The construction of autobiographical memories in the self-memory system. Psychol. Rev..

[17] Rubin D.C. (2006). The basic-systems model of episodic memory. Perspect. Psychol. Sci..

[18] Conway M.A., Singer J.A., Tagini A. (2004). The self and autobiographical memory: Correspondence and coherence. Soc. Cogn..

[19] Rathbone C.J., Moulin C.J.A., Conway M.A. (2008). Self-centered memories: The reminiscence bump and the self. Mem. Cognit..

[20] Conway M.A. (2009). Episodic memories. Neuropsychologia.

[21] McHan K., Johnston-Taylor E., Piscopo B., Abate E., Dehom S. (2022). Nursing values and moral identity in baccalaureate nursing students. J. Prof. Nurs..

[22] Charbonnier-Voirin A., Roussel P. (2012). Adaptive performance: A new scale to measure individual performance in organizations. Can. J. Adm. Sci..

[23] Litz B.T., Contractor A., Rhodes C., Dondanville K., Jordan A., Resick P., Foa E.B., Young-McCaughan S., Mintz J., Yarvis J.S. (2018). Distinct trauma types in military service members seeking treatment for posttraumatic stress disorder. J. Trauma. Stress.

[24] Litz B.T., Kerig P.K. (2019). Introduction to the Special Issue on Moral Injury: Conceptual Challenges, Methodological Issues, and Clinical Applications. J. Trauma. Stress.

[25] Philippe F.L. (2022). Episodic memories as proxy or independent representations: A theoretical review and an empirical test of distinct episodic memories on work outcomes. New Ideas Psychol..

[26] Milyavskaya M., Philippe F.L., Koestner R. (2013). Psychological need satisfaction across levels of experience: Their organization and contribution to general well-being. J. Res. Pers..

[27] Philippe F.L., Lopes M., Houlfort N., Fernet C. (2019). Work-related episodic memories can increase or decrease motivation and psychological health at work. Work Stress.

[28] Conway M., Meares K., Standart S. (2004). Images and goals. Memory.

[29] Smyth J., True N., Souto J. (2001). Effects of writing about traumatic experiences: The necessity for narrative structuring. J. Soc. Clin. Psychol..

[30] Philippe F.L., Bernard-Desrosiers L. (2017). The odyssey of episodic memories: Identifying the paths and processes through which they contribute to well-being. J. Pers..

[31] Philippe F.L., Bouizegarene N., Guilbault V., Rajotte G., Houle I. (2015). The chicken or the egg? Systematic investigation of the effect of order of administration of memory questionnaires and well-being scales. Memory.

[32] Philippe F.L., Koestner R., Beaulieu-Pelletier G., Lecours S. (2011). The role of need satisfaction as a distinct and basic psychological component of autobiographical memories: A look at well-being. J. Pers..

[33] Philippe F.L., Koestner R., Beaulieu-Pelletier G., Lecours S., Lekes N. (2012). The role of episodic memories in current and future well-being. Pers. Soc. Psychol. Bull..

[34] Philippe F.L., Koestner R., Lecours S., Beaulieu-Pelletier G., Bois K. (2011). The role of autobiographical memory networks in the experience of negative emotions: How our remembered past elicits our current feelings. Emotion.

[35] Philippe F.L., Koestner R., Lekes N. (2013). On the directive function of episodic memories in people’s lives: A look at romantic relationships. J. Pers. Soc. Psychol..

[36] Ryan R.M., Deci E.L. (2017). Self-Determination Theory: Basic Psychological Needs in Motivation, Development, and Wellness.

[37] Tay L., Diener E. (2011). Needs and subjective well-being around the world. J. Pers. Soc. Psychol..

[38] Dweck C.S. (2017). From needs to goals and representations: Foundations for a unified theory of motivation, personality, and development. Psychol. Rev..

[39] Klein S.B., Loftus J., Trafton J.G., Fuhrman R.W. (1992). Use of exemplars and abstractions in trait judgments: A model of trait knowledge about the self and others. J. Pers. Soc. Psychol..

[40] Ramos A.M., Barlem E.L.D., Barlem J.G.T., Rocha L.P., Dalmolin G.d.L., Figueira A.B. (2017). Cross-cultural adaptation and validation of the Moral Distress Scale-Revised for nurses. Rev. Bras. Enferm..

[41] Bartholdson C., Molewijk B., Lützén K., Blomgren K., Pergert P. (2018). Ethics case reflection sessions: Enablers and barriers. Nurs. Ethics.

[42] Yeung S.K., Yay T., Feldman G. (2021). Action and Inaction in Moral Judgments and Decisions: Meta-Analysis of Omission Bias Omission-Commission Asymmetries. Pers. Soc. Psychol. Bull..

[43] Fruet I.M.A., Dalmolin G.d.L., Barlem E.L.D., Silva R.M.d., Andolhe R. (2018). Aplicabilidade da Moral Distress Scale adaptada no cenário da enfermagem em hemato-oncologia. Rev. Gaucha Enferm..

[44] Soleimani M.A., Sharif S.P., Yaghoobzadeh A., Panarello B. (2019). Psychometric evaluation of the Moral Distress Scale-Revised among Iranian Nurses. Nurs. Ethics.

[45] Abdolmaleki M., Lakdizaji S., Ghahramanian A., Allahbakhshian A., Behshid M. (2019). Relationship between autonomy and moral distress in emergency nurses. Indian J. Med. Ethics.

[46] Maslach C., Jackson S.E., Leiter M.P. (1996). MBI: Maslach Burnout Inventory.

[47] Litam S.D.A., Balkin R.S. (2021). Moral injury in health-care workers during COVID-19 pandemic. Traumatology.

[48] Wang Z., Harold K.G., Tong Y., Wen J., Sui M., Liu H., Zaben F.A., Liu G. (2022). Moral injury in Chinese health professionals during the COVID-19 pandemic. Psychol. Trauma.

[49] Norman S.B., Feingold J.H., Kaye-Kauderer H., Kaplan C.A., Hurtado A., Kachadourian L., Feder A., Murrough J.W., Charnie D., Southwick S.M. (2021). Moral distress in frontline healthcare workers in the initial epicenter of the COVID-19 pandemic in the United States: Relationship to PTSD symptoms, burnout, and psychosocial functioning. Depress. Anxiety.

[50] Galanis P., Vraka I., Fragkou D., Bilali A., Kaitelidou D. (2021). Nurses’ burnout and associated risk factors during the COVID-19 pandemic: A systematic review and meta-analysis. J. Adv. Nurs..

[51] Gómez-Urquiza J.L., Vargas C., De la Fuente E.I., Fernández-Castillo R., Cañadas-De la Fuente G.A. (2017). Age as a risk factor for burnout syndrome in nursing professionals: A meta-analytic study. Res. Nurs. Health.

[52] Griffeth R.W., Hom P.W., Gaertner S. (2000). A meta-analysis of antecedents and correlates of employee turnover: Update, moderator tests, and research implications for the next millennium. J. Manag..

[53] Tett R.P., Meyer J.P. (1993). Job satisfaction, organizational commitment, turnover intention, and turnover: Path analyses based on meta-analytic findings. Pers. Psychol..

[54] Falatah R. (2021). The Impact of the Coronavirus Disease (COVID-19) Pandemic on Nurses’ Turnover Intention: An Integrative Review. Nurs. Rep..

[55] Nikkhah-Farkhani Z., Piotrowski A. (2020). Nurses’ turnover intention—A comparative study between Iran and Poland. Med. Pract..

[56] Kim Y.-J., Lee S.-Y., Cho J.-H. (2020). A Study on the Job Retention Intention of Nurses Based on Social Support in the COVID-19 Situation. Sustainability.

[57] Irshad M., Khattak S.A., Hassan M.M., Majeed M., Bashir S. (2020). Withdrawn: How perceived threat of COVID-19 causes turnover intention among Pakistani nurses: A moderation and mediation analysis. Int. J. Ment. Health Nurs..

[58] Chin E.G., Clubbs B.H. (2022). Pandemic Issues: Faculty Value Alignment and Burnout. J. Educ. Res. Pract..

[59] Albougami A.S., Almazan J.U., Cruz J.P., Alquwez N., Alamri M.S., Adolfo C., Roque M.Y. (2020). Factors Affecting Nurses’ Intention to Leave Their Current Jobs in Saudi Arabia. Int. J. Health Sci..

[60] De Oliveira D.R., Griep R.H., Portela L.F., Rotenberg L. (2017). Intention to leave profession, psychosocial environment, and self-rated health among registered nurses from large hospitals in Brazil: A cross-sectional study. BMC Health Serv. Res..

[61] Pélissier C., Charbotel B., Fassier J.B., Fort E., Fontana L. (2018). Nurses’ Occupational and Medical Risks Factors of Leaving the Profession in Nursing Homes. Int. J. Environ. Res. Public Health.

[62] Yáñez J.A., Jahanshahi A.A., Alvarez-Risco A., Li J., Zhang S.X. (2020). Anxiety, Distress, and Turnover Intention of Healthcare Workers in Peru by Their Distance to the Epicenter during the COVID-19 Crisis. Am. J. Trop. Med. Hyg..

[63] Dascalu S., Geambasu O., Raiu V.C., Azoicai D., Popovici D.E., Apetrei C. (2021). COVID-19 in Romania: What Went Wrong?. Front. Public Health.

[64] Greenberg N., Docherty M., Gnanapragasam S., Wessely S. (2020). Managing mental health challenges faced by healthcare workers during COVID-19 pandemic. BMJ.

[65] Bria M., Spânu F., Băban A., Dumitraşcu D.L. (2014). Maslach burnout inventory–general survey: Factorial validity and invariance among Romanian healthcare professionals. Burn. Res..

[66] Cammann C., Fichman M., Jenkins D., Klesh J. (1979). The Michigan Organizational Assessment Questionnaire.

[67] Van den Broeck A., Vansteenkiste M., De Witte H., Soenens B., Lens W. (2010). Capturing autonomy, competence, and relatedness at work: Construction and initial validation of the Work-related Basic Need Satisfaction scale. J. Occup. Organ. Psychol..

[68] Borges L.M., Holliday R., Barnes S.M., Bahraini N.H., Kinney A., Forster J.E., Brenner L.A. (2021). A longitudinal analysis of the role of potentially morally injurious events on COVID-19-related psychosocial functioning among healthcare providers. PLoS ONE.

[69] Deci E.L., Ryan R.M., Gagne M., Leone D.R., Usunov J., Kornazheva B.P. (2001). Need satisfaction, motivation, and well-being in the work organizations of a former eastern bloc country: A cross-cultural study of self-determination. Pers. Soc. Psychol. Bull..

[70] Gagne M. (2003). The role of autonomy support and autonomy orientation in prosocial behavior engagement. Motiv. Emot..

[71] Hagger M.S., Chatzisarantis N.L.D., Harris J. (2006). From Psychological Need Satisfaction to Intentional Behavior: Testing a Motivational Sequence in Two Behavioral Contexts. Pers. Soc. Psychol. Bull..

[72] La Guardia J.G., Ryan R.M., Couchman C.E., Deci E.L. (2000). Within-person variation in security of attachment: A self-determination theory perspective on attachment, need fulfillment, and well-being. J. Pers Soc. Psychol..

[73] Berntsen D. (2010). The unbidden past: Involuntary autobiographical memories as a basic mode of remembering. Curr. Dir. Psychol. Sci..

[74] Johannessen K.B., Berntsen D. (2010). Current concerns in involuntary and voluntary autobiographical memories. Conscious. Cogn..

[75] Hall N.M., Berntsen D. (2008). The effect of emotional stress on involuntary and voluntary conscious memories. Memory.

[76] Johnson M.K., Foley M.A., Suengas A.G., Raye C.L. (1988). Phenomenal characteristics of memories for perceived and imagined autobiographical events. J. Exp. Psychol..

[77] Rubin D.C., Schrauf R.W., Greenberg D.L. (2003). Belief and recollection of autobiographical memories. Mem. Cogn..

[78] Huang S., Stanley M.L., De Brigard F. (2020). The phenomenology of remembering our moral transgressions. Mem. Cognit..

[79] Brewin C.R., Dalgleish T., Joseph S. (1996). A dual representation theory of post- traumatic stress disorder. Psychol. Rev..

[80] Krans J., Naring G., Becker E.S., Holmes E.A. (2009). Intrusive trauma memory: A review and functional analysis. Appl. Cogn. Psychol..

[81] Pillemer D.B., Winograd E., Neisser U. (1992). Remembering personal circumstances: A functional analysis. Affect and Accuracy in Recall: Studies of “Flashbulb” Memories.

[82] Pillemer D.B. (2003). Directive functions of autobiographical memory: The guiding power of the specific episode. Memory.

[83] Gibbons S.W., Shafer M., Hickling E.J., Ramsey G. (2013). How do deployed health care providers experience moral injury?. Narrat. Inq. Bioeth..

[84] Haight W., Sugrue E., Calhoun M., Black J. (2017). Everyday coping with moral injury: The perspectives of professionals and parents involved with child protection services. Child. Youth Serv. Rev..

[85] Rubin D.C., Berntsen D., Bohni M.K. (2008). A memory-based model of posttraumatic stress disorder: Evaluating basic assumptions underlying the PTSD diagnosis. Psychol. Rev..

[86] Helion C., Helzer E.G., Kim S., Pizarro D.A. (2020). Asymmetric memory for harming versus being harmed. J. Exp. Psychol. Gen..

[87] French L., Hanna P., Huckle C. (2022). “If I die, they do not care”: U.K. National Health Service staff experiences of betrayal-based moral injury during COVID-19. Psychol. Trauma.

[88] Aldrup K., Klusmann U., Lüdtke O. (2017). Does basic need satisfaction mediate the link between stress exposure and well-being? A diary study among beginning teachers. Learn. Instr..

[89] Maslach C., Schaufeli W.B., Leiter M.P. (2001). Job burnout. Annu. Rev. Psychol..

[90] Gillet N., Colombat P., Michinov E., Pronost A.M., Fouquereau E. (2013). Procedural justice, supervisor autonomy support, work satisfaction, organizational identification and job performance: The mediating role of need satisfaction and perceived organizational support. J. Adv. Nurs..

[91] Gillet N., Fouquereau E., Coillot H., Cougot B., Moret L., Dupont S., Bonnetain F., Colombat P. (2018). The effects of work factors on nurses’ job satisfaction, quality of care and turnover intentions in oncology. J. Adv. Nurs..

[92] Maillet S., Read E. (2021). Work Environment Characteristics and Emotional Intelligence as Correlates of Nurses’ Compassion Satisfaction and Compassion Fatigue: A Cross-Sectional Survey Study. Nurs. Rep..

[93] Manzano García G., Ayala Calvo J.C. (2021). The threat of COVID-19 and its influence on nursing staff burnout. J. Adv. Nurs..

[94] Slåtten T., Mutonyi B.R., Lien G. (2020). The impact of individual creativity, psychological capital, and leadership autonomy support on hospital employees’ innovative behaviour. BMC Health Serv. Res..

[95] Xu Y., Liu D., Tang D.S. (2022). Decent work and innovative work behaviour: Mediating roles of work engagement, intrinsic motivation and job self-efficacy. Creat. Innov. Manag..

[96] Lorber M., Treven S., Mumel D. (2020). Well-Being and Satisfaction of Nurses in Slovenian Hospitals: A Cross-Sectional Study. Slov. J. Public Health.

[97] Onyishi I.E., Enwereuzor I.K., Ogbonna M.N., Ugwu F.O., Amazue L.O. (2019). Role of Career Satisfaction in Basic Psychological Needs Satisfaction and Career Commitment of Nurses in Nigeria: A Self-Determination Theory Perspective. J. Nurs. Scholarsh..

[98] Gaudet C.M., Sowers K.M., Nugent W.R., Boriskin J.A. (2016). A review of PTSD and shame in military veterans. J. Hum. Behav. Soc..

[99] Nazarov A., Jetly R., McNeely H., Kiang M., Lanius R., McKinnon M.C. (2015). Role of morality in the experience of guilt and shame within the Armed Forces. Acta Psychiatr. Scand..

[100] Farnsworth J.K., Drescher K.D., Evans W., Walser R.D. (2017). A functional approach to understanding and treating military-related moral injury. J. Context. Behav. Sci..

[101] Maguen S., Burkman K., Madden E., Dinh J., Bosch J., Keyser J., Schmitz M., Neylan T.C. (2017). Impact of killing in war: A randomized, controlled pilot trial. J. Clin. Psychol..

[102] Yeterian J.D., Berke D.S., Litz B.T. (2017). Psychosocial rehabilitation after war trauma with adaptive disclosure: Design and rationale of a comparative efficacy trial. Contemp. Clin. Trials.

[103] Gray K., Waytz A., Young L. (2012). The moral dyad: A fundamental template unifying moral judgment. Psychol. Inq..

[104] Brüggemann A.J., Wijma B., Swahnberg K. (2012). Abuse in health care: A concept analysis. Scand. J. Caring Sci..

